# A Meta-Analysis of Parental Smoking and the Risk of Childhood Brain Tumors

**DOI:** 10.1371/journal.pone.0102910

**Published:** 2014-07-24

**Authors:** Yi Huang, Jianrong Huang, Huan Lan, GuanYan Zhao, ChunZhen Huang

**Affiliations:** Department of Neurosurgery, Guangxi Minzu Hospital, Nanning, Guangxi Province, China; National Health Research Institutes, Taiwan

## Abstract

**Objective:**

Previous studies regarding the association between parental smoking and the risk of childhood brain tumors (CBT) have reported inconsistent results. We performed a meta-analysis to summarize evidence on this association and to quantify the potential dose-response relationship.

**Methods:**

A systematic literature search was conducted in the Medline and Embase databases. The summary relative risks (RRs) with 95% confidence intervals (CIs) were calculated. Dose–response meta-analysis was also performed for studies that reported categorical risk estimates for a series of smoking exposure levels.

**Results:**

A total of 17 studies fulfilled the inclusion criteria. In the meta-analyses, the summary RRs (95% CIs) of CBT for maternal smoking during pregnancy, paternal smoking during pregnancy, maternal smoking before pregnancy, and paternal smoking before pregnancy were 0.96 (0.86–1.07), 1.09 (0.97–1.22), 0.93 (0.85–1.00), and 1.09 (1.00–1.20), respectively. Dose-response meta-analysis also showed no significant association between parental smoking and the risk of CBT.

**Conclusions:**

Findings from our meta-analysis indicate that parental smoking may not be associated with a risk of CBT.

## Introduction

Childhood brain tumors (CBT) are one of the most common types of cancers in infants and children (behind hematological malignancies) and they account for approximately 20 to 25% of total primary pediatric tumor diagnoses [1]. Their 5-year survival ranges from >90% to <10% for various histological subtypes [2]. A small percentage of these tumors are found in the setting of an identifiable cancer predisposition syndrome, such as neurofibromatosis and melanoma–astrocytoma syndrome [3]. However, for most sporadic cases, little is known about the genetic or environmental etiologies.

Cigarette smoking is a major cause of illness and death worldwide. The first phase of Global Adult Tobacco Survey (GATS) reported that a high percentage of men smoke, women begin smoking early, and few successfully quit smoking [4]. It has been hypothesized that some cancers may begin during the early stages of fetal development [5]. The exposure to environmental cigarette smoke during pregnancy could lead to DNA mutations and cytogenetic damage and has been shown to act as a transplacental carcinogen in animal studies [6]–[8]. Increased levels of carcinogenic tobacco-specific nitrosamines could be detected in the urine samples of newborns and the amniotic fluid in early pregnancy of parents who smoked cigarettes during pregnancy [9]–[11]. Therefore, parental smoking, which is relatively frequent, may play a role in tumorigenesis of CBT and require further exploration.

However, epidemiological studies on a possible association between parental smoking and the risk of CBT have provided no definitive answers. Overall, the published literature remains inconclusive and inconsistent. For example, a Sweden cohort study and an Italian case–control study suggested a positive association between maternal smoking during pregnancy and the risk of CBT [12], [13], whereas a UK case–control study reported a negative association between them [14]. Because of the relatively small number of cases included in the individual studies, we performed a comprehensive meta-analysis to summarize the evidence on whether parental smoking is associated with the risk of CBT.

## Methods

### Search Strategy

We conducted a literature search (up to January 2014) of Medline and Embase for studies examining the association between parental smoking and the risk of CBT. The search terms were (case-control OR cohort OR epidemiolog*) AND (cancer OR carcinoma OR neoplasms OR tumor OR tumour) AND (brain OR cerebral OR intracranial OR central nervous system OR glioma OR glioblastoma OR astrocytoma OR craniopharyngioma OR medulloblastoma OR PNET OR ependymomas) AND (smoke OR smoking OR cigarette OR tobacco) for Medline. Similar search terms were used for Embase. We searched articles published in any language and scrutinized references cited by these studies to identify further pertinent studies. This meta-analysis followed the standard criteria for conducting meta-analyses of observational studies and reporting the results [15].

### Study selection

We applied the following inclusion criteria: the study used a cohort or case-control design, the exposure of interest was parental smoking, the outcome was CBT and the investigators provided the minimum information necessary to estimate the relative risk (RR) with 95% confidence intervals (CIs). We excluded animal studies, cross sectional studies, reviews, editorials, commentaries, and letters without sufficient data. If data sets overlapped or were duplicated, the most up-to-date or comprehensive information was included.

### Data extraction

Data were extracted independently by two authors and any disagreements were resolved by consensus. The following information was recorded: the first author's last name, year of publication, study location, types of CBT, sample size (cases and controls or cohort size), exposure (smoking levels for each category) assessment method, outcome (CBT) ascertainment, covariates adjusted for in the analysis, and RR estimates with corresponding 95% CIs for each category. If available, we used the RRs that reflected the greatest degree of control for potential confounders.

### Statistical analysis

RR was used as a measure of the association between parental smoking and the risk of CBT. For case-control studies, the odds ratio (OR) was used as a surrogate measure of the corresponding RR. Because the absolute risk of CBT is low, the OR approximates the RR [16].

Summary RRs (95% CI) were calculated by combining the study-specific RR estimates with the DerSimonian Laird method (random effects model) [17]. In the dose–response meta-analysis, we used the method described by Greenland [18] and Orsini [19] to calculate the trend from the correlated estimates for relative risk across smoking categories. The midpoint of the upper and lower boundaries in each category was assigned as the corresponding dose of consumption. If the highest category was open ended, we assumed the width of the interval to be the same as the width in the preceding category. We explored a potential curvilinear relationship between parental smoking and the risk of CBT using restricted cubic splines with three knots at percentiles 25%, 50%, and 75% of the distribution [20]. A *P* value for nonlinearity was calculated by testing the null hypothesis that the coefficient of the second spline was equal to 0.

We calculated the Q statistic (*P*<0.1 was considered to be indicative of statistically significant heterogeneity) and I^2^ statistic (I^2^<25% no heterogeneity; I^2^ = 25% to 50% low heterogeneity; I^2^ = 50% to 75% moderate heterogeneity; I^2^>75% large or extreme heterogeneity) [21] to assess heterogeneity across studies. We also conducted analyses stratified by study design, study location, histological subtype, number of cases and publication year. Influence analysis was performed, in which the summary estimates were computed after the omission of each study in turn. An estimation of potential publication bias was evaluated by Begg's adjusted rank correlation test [22] and Egger's regression asymmetry test [23]. All statistical tests were conducted with the STATA software (version 11.0; Stata Corporation, College Station, Texas). *P* values were two sided with a significance level of 0.05.

## Results

### Literature search

**Figure 1:**
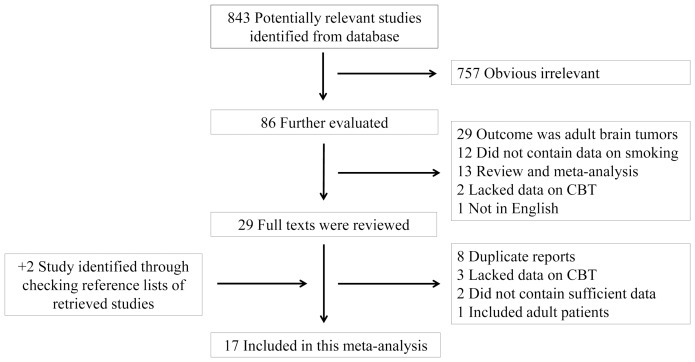
Flowchart of study assessment and selection.

A flow diagram of our literature search and study selection is shown in Figure 1. We identified 843 unique citations from Medline and Embase. After excluding studies that did not meet our inclusion criteria, 29 remaining articles appeared to be potentially relevant for this meta-analysis. After evaluating the full texts of these publications, we further excluded 14 articles for the following reasons: duplicate reports from the same study population (n = 8); no data on CBT (n = 3); lack of sufficient data to calculate RR and 95% CIs (n = 2); adult patients included (n = 1). A manual search of references cited by these papers yielded 2 new eligible articles. Therefore, we finally included 17 articles [12]–[14], [24]–[37] in the meta-analysis.

### Study Characteristics

The 17 studies were published between 1986 and 2013, involving a total of 5,098 cases. Of these studies, 6 were conducted in North America [25], [32]–[36], 6 in Europe [12]–[14], [27], [29], [37], 2 in Australia [24], [26], 2 in China [30], [31] and 1 was multi-centered [28]. 2 studies were cohort studies [12], [26], and 15 were case-control studies [13], [14], [24], [25], [27]–[37]. Of the 17 studies, 16 had information on maternal smoking during pregnancy [12]–[14], [24]–[30], [32]–[37], 7 had data on maternal smoking before pregnancy [13], [14], [24], [28], [30], [33], [34], 9 on paternal smoking during pregnancy [24], [25], [27], [29], [30], [32]–[34], [36], and 7 on paternal smoking before pregnancy [13], [14], [24], [28], [30], [31], [33]. 8 studies considered all brain cancers together only [24]–[26], [30], [31], [34], [36], [37], 1 considered astrocytoma only [35], and 8 considered several subtypes of CBT and provided separate analyses for these cancer subtypes [12]–[14], [27]–[29], [32], [33]. In 12 of the 15 incident case-control studies, controls were matched for age and sex [13], [14], [24], [25], [27]–[31], [33], [34], [36]. The numbers of cases and controls or cohort, types of CBT, exposure assessment method, and outcome ascertainment were shown in Table 1.

**Table 1:** Study characteristics of published cohort and case–control studies on parental smoking and the risk of childhood brain tumors.

First author	Year	Design	Region	Exposure assessment	Outcome ascertainment	Diagnosis criteria	Age	Matched factors	Types of CBT	Cases	Controls or cohort
Barrington-Trimis et al	2013	PCC	USA	In-person interview	SEER registries	ICD-O-1	≤10	Age, sex, center	Astroglial	97	285
									PNET	55	
									Others	50	
Milne et al	2013	PCC	Australia	Mailed questionnaire	Pediatric oncology center	NR	≤15	Age, sex, state	Gliomas	170	941
									Embryonal tumors	71	
									Germ cell tumors	20	
									Ependymomas	22	
									Others	19	
Stavrou et al	2009	Cohort	Australia	Midwives Data Collection	Central Cancer Registry	ICD-O-3	≤12	-	Total CBT	143	1,045,966
Plichart et al	2008	PCC	France	Telephone interview	French National Registry	ICD-O-3	≤15	Age, sex	Embryonal tumors	100	1,681
									Ependymomas	33	
									Astrocytomas	26	
									Others	50	
Brooks et al	2004	Cohort	Sweden	Swedish Birth Register	Swedish Cancer Register	ICD7	NR	-	Ependymoma	51	1,441,942
									Astrocytoma	220	
									Medulloblastoma	62	
									Others	147	
Pang et al	2003	PCC	UK	Interview	Pediatric oncology units	ICD-O-2	≤15	Age, sex, residence area	Total CBT	635	6,987
Filippini et al	2002	PCC	Multicenter	Interviewed in person	Cancer registries	ICD-O-2	≤19	Age, sex	Astroglial tumor	623	2,223
									PNET	259	
									Others	336	
Schuz et al	2001	PCC	Germany	Questionnaire, telephone interview	Childhood Cancer Registry	NR	≤15	Age, sex	Astrocytoma	119	2,458
									Medulloblastoma	112	
									Ependymoma	50	
									Others	185	
Filippini et al	2000	PCC	Italy	Telephone interview	Hospital records	ICD-9	≤15	Age, sex, residence area	Astroglial tumours	115	502
									PNET	37	
									Others	82	
Hu et al	2000	HCC	China	Interview	Six major hospitals	NR	≤18	Age, sex, residence area	Astrocytoma	21	246
									Medulloepithelioma	13	
									Craniopharyngioma	11	
									Others	38	
Ji et al	1997	PCC	China	Direct interview	Cancer Registry	ICD-9	≤15	Age, sex	Total CBT	107	107
Bunin et al	1994	PCC	USA and Canada	Telephone interview	Children's Cancer Group	NR	≤6	Age, race, residence area	Astrocytoma	155	321
									PNET	166	
Gold et al	1993	PCC	USA	Structured interview	SEER program registries	NR	≤18	Age, sex, mother's race	Astrocytoma	152	1,083
									Medulloblastoma	60	
									Others	126	
John et al	1991	PCC	USA	Structured interviews	Cancer Registry	NR	≤14	Age, sex, residence area	Total CBT	48	196
Kuitjen et al	1990	PCC	USA	Telephone interview	Tumor registries	NR	≤15	Age, race, residence area	Astrocytoma	163	163
Howe et al	1989	PCC	Canada	In-person interview	Hospital records	NR	≤18	Age, sex	Astrocytoma	21	138
									Medulloblastoma	24	
									Ependymoma	10	
									Others	19	
Stjernfeldt et al	1986	PCC	Sweden	NR	NR	NR	≤16	NR	Total CBT	43	NR

PCC population based case-control, HCC hospital based case-control, NR not reported.

### Quantitative synthesis

**Figure 2:**
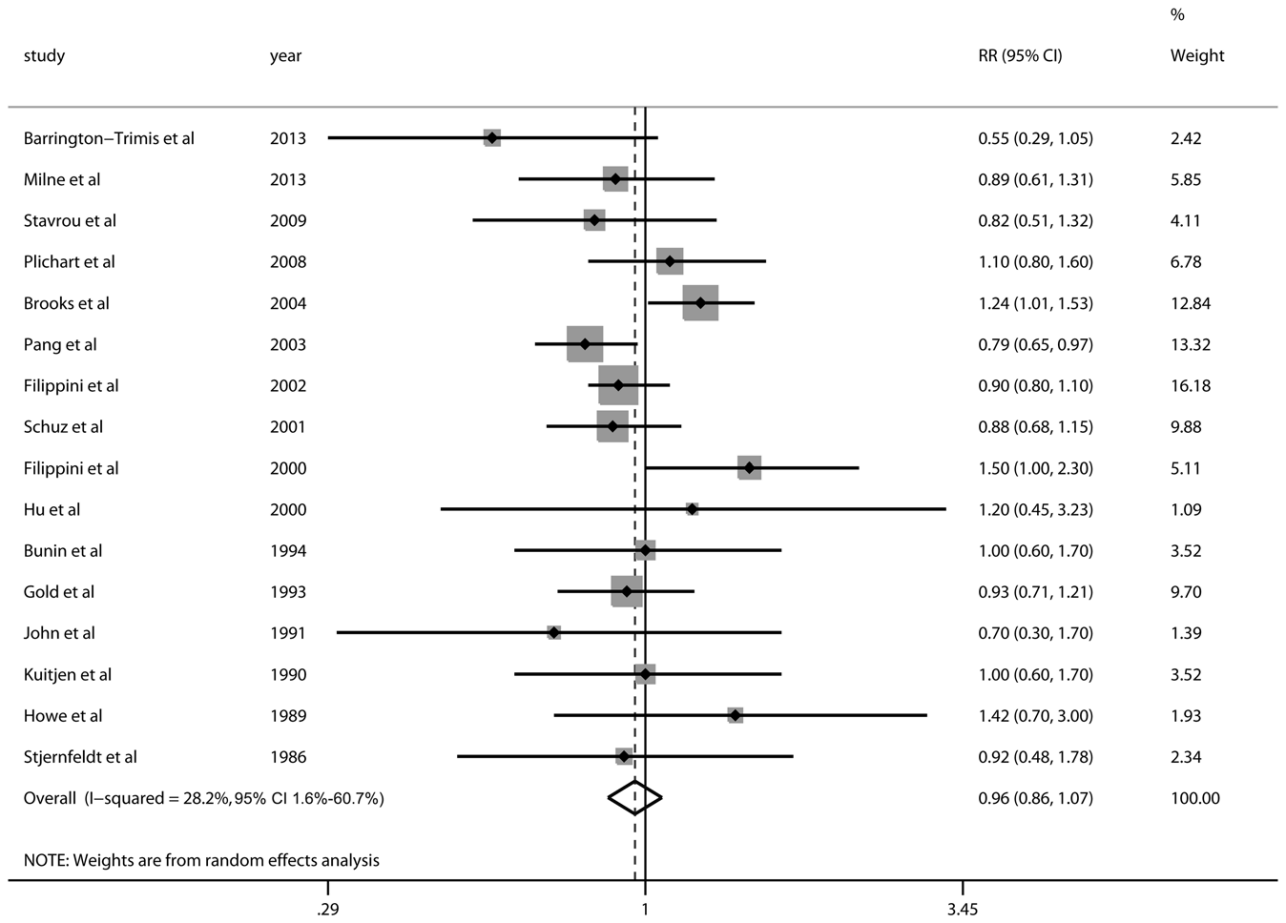
Forest plot of maternal smoking during pregnancy and the risk of CBT.

The pooled RRs between parental smoking and the risk of CBT were not statistically significant and close to unity (RR = 0.96, 95% CI 0.86–1.07 for maternal smoking during pregnancy; RR = 1.09, 95% CI 0.97–1.22 for paternal smoking during pregnancy; RR = 0.93, 95% CI 0.85–1.00 for maternal smoking before pregnancy; RR = 1.09, 95% CI 1.00–1.20 for paternal smoking before pregnancy) (Figures 2 and S1).

In the stratified analysis by study region, histological subtype, number of cases, and publication year, no significant associations were observed in any of the categories (Tables 2–5).

**Table 2:** Results of subgroup analyses of the association between maternal smoking during pregnancy and the risk of childhood brain tumors.

				Heterogeneity test	
Variables	Study	RR (95% CI)	*P* *	Q	I^2^ (95%CI) (%)
Total	16 (12–14,24–30,32–37)	0.96 (0.86–1.07)		20.88	28.2 (1.6–60.7)
Study design			0.072		
Cohort	2 (12,26)	1.07 (0.72–1.58)		2.44	59.0 (-)
Case–control	14 (13,14,24,25,27–30,32–37)	0.92 (0.84–1.00)		13.41	3.1 (0.0–56.4)
Geographical region			0.816		
North America	6 (25,32–36)	0.92 (0.76–1.11)		4.41	0.0 (0.0–74.62)
Europe	6 (12–14,27,29,37)	1.03 (0.84–1.27)		14.35	65.2 (16.3–85.5)
Australia	2 (24,26)	0.86 (0.64–1.16)		0.07	0.0 (-)
China	1 (30)	1.20 (0.45–3.23)		-	-
Histological subtype			-		
PNET	8 (12–14,27–29,32,33)	0.89 (0.74–1.06)		6.58	0.0 (0.0–67.6)
Astrocytomas	9 (12–14,27–29,32,33,35)	1.05 (0.93–1.18)		7.69	0.0 (0.0–64.8)
Ependymomas	4 (12,14,27,29)	1.09 (0.72–1.66)		4.22	28.9 (0.0–73.8)
No of cases			0.492		
≤ 300	9 (13,25–27,30,34–37)	1.02 (0.83–1.25)		9.53	16.1 (0.0–58.1)
>300	7 (12,14,24,28,29,32,33)	0.94 (0.83–1.06)		10.38	42.2 (0.0–75.7)
Publication year			0.289		
≤2000	8 (13,30,32–37)	1.05 (0.88–1.24)		5.41	0.0 (0.0–67.6)
>2000	8 (12,14,24–29)	0.92 (0.80–1.06)		14.04	50.1 (0.0–77.7)

* *P* for heterogeneity of the stratum-specific summary RRs.

**Table 3:** Results of subgroup analyses of the association between paternal smoking during pregnancy and the risk of childhood brain tumors

				Heterogeneity test	
Variables	Number	RR (95% CI)	*P* *	Q	I^2^ (95%CI) (%)
Total	9 (24,25,27,29,30,32–34,36)	1.09 (0.97–1.22)		2.35	0.0 (0.0–64.8)
Geographical region			0.892		
North America	5 (25,32–34,36)	1.03 (0.85–1.25)		1.45	0.0 (0.0–79.2)
Europe	2 (27,29)	1.13 (0.96–1.34)		0.23	0.0 (-)
Australia	1 (24)	1.04 (0.74–1.46)		-	-
China	1 (30)	1.17 (0.67–2.04)		-	-
Histological subtype			-		
PNET	4 (27,29,32,33)	1.10 (0.88–1.37)		0.50	0.0 (0.0–84.7)
Astrocytomas	4 (27,29,32,33)	1.13 (0.79–1.61)		6.27	52.1 (0.0–84.2)
Ependymomas	2 (27,29)	1.48 (0.99–2.20)		0.07	0.0 (-)
No of cases			0.160		
≤ 300	5 (25,27,30,34,36)	1.18 (0.96–1.45)		0.86	0.0 (0.0–79.2)
>300	4 (24,29,32,33)	1.04 (0.91–1.20)		0.56	0.0 (0.0–84.7)
Publication year			0.673		
≤2000	5 (30,32–34,36)	1.05 (0.86–1.28)		1.63	0.0 (0.0–79.2)
>2000	4 (24,25,27,29)	1.11 (0.96–1.28)		0.51	0.0 (0.0–84.7)

* *P* for heterogeneity of the stratum-specific summary RRs.

**Table 4:** Results of subgroup analyses of the association between maternal smoking before pregnancy and the risk of childhood brain tumors.

				Heterogeneity test	
Variables	Study	RR (95% CI)	*P* *	Q	I^2^ (95%CI) (%)
Total	7 (13,14,24,28,30,33,34)	0.93 (0.85–1.00)		3.23	0.0 (0.0–70.8)
Geographical region			0.839		
North America	2 (33,34)	0.89 (0.69–1.15)		0.00	0.0 (-)
Europe	2 (13,14)	1.02 (0.79–1.31)		2.07	51.6 (-)
Australia	1 (24)	0.99 (0.70–1.40)		-	-
China	1 (30)	0.62 (0.10–3.80)		-	-
Histological subtype			-		
PNET	3 (14,28,33)	0.87 (0.69–1.09)		0.59	0.0 (0.0–89.6)
Astrocytomas	3 (14,28,33)	0.91 (0.80–1.03)		0.04	0.0 (0.0–89.6)
Ependymomas	1 (14)	0.73 (0.40–1.35)		-	-
No of cases			0.427		
≤300	3 (13,30,34)	1.14 (0.85–1.53)		0.84	0.0 (0.0–89.6)
>300	4 (14,24,28,33)	0.91 (0.83–0.99)		0.31	0.0 (0.0–84.7)
Publication year			0.962		
≤ 2000	4 (13,30,33,34)	0.99 (0.82–1.21)		2.32	0.0 (0.0–84.7)
>2000	3 (14,24,28)	0.91 (0.83–1.00)		0.28	0.0 (0.0–89.6)

* *P* for heterogeneity of the stratum-specific summary RRs.

**Table 5:** Results of subgroup analyses of the association between paternal smoking before pregnancy and the risk of childhood brain tumors.

				Heterogeneity test	
Variables	Number	RR (95% CI)	*P* *	Q	I^2^ (95%CI) (%)
Total	7 (13,14,24,28,30,31,33)	1.09 (1.00–1.20)		3.29	0.0 (0.0–70.8)
Geographical region			0.605		
North America	1 (33)	1.08 (0.83–1.41)		-	-
Europe	2 (13,14)	1.08 (0.93–1.25)		0.53	0.0 (-)
Australia	1 (24)	0.99 (0.71–1.38)		-	-
China	2 (30,31)	1.42 (0.90–2.24)		1.07	6.3 (-)
Histological subtype			-		
PNET	3 (14,28,33)	0.94 (0.77–1.16)		1.50	0.0 (0.0–89.6)
Astrocytomas	3 (14,28,33)	1.11 (0.95–1.28)		1.37	0.0 (0.0–89.6)
Ependymomas	1 (14)	1.03 (0.59–1.78)		-	-
No of cases			0.130		
≤ 300	3 (13,30,31)	1.27 (0.98–1.64)		1.42	0.0 (0.0–89.6)
>300	4 (14,24,28,33)	1.07 (0.97–1.18)		0.41	0.0 (0.0–84.7)
Publication year			0.151		
≤ 2000	4 (13,30,31,33)	1.17 (0.98–1.41)		2.15	0.0 (0.0–84.7)
>2000	3 (14,24,28)	1.07 (0.96–1.19)		0.40	0.0 (0.0–89.6)

* *P* for heterogeneity of the stratum-specific summary RRs.

### Dose-response analysis

**Figure 3:**
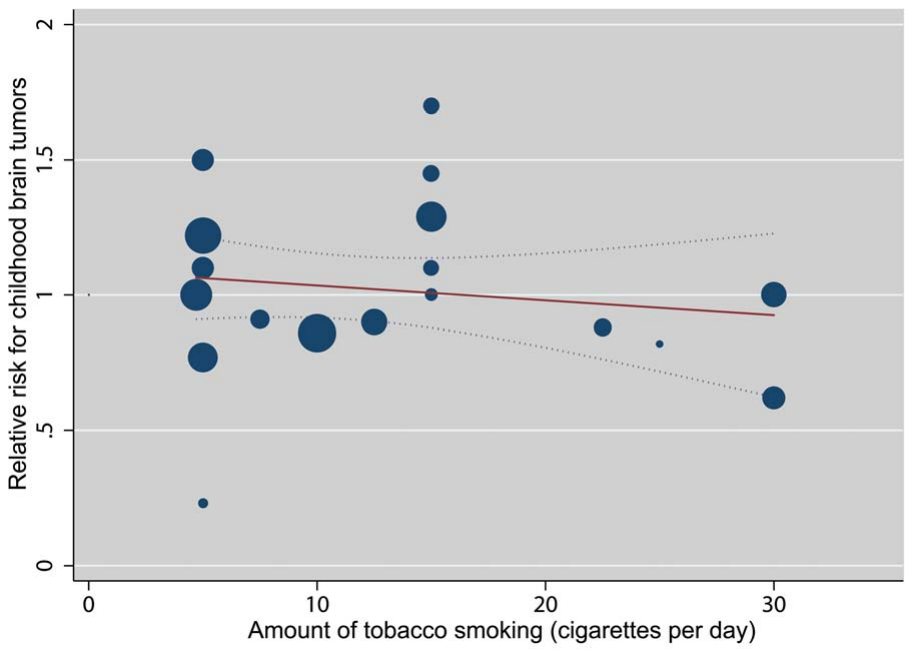
Dose-response analysis of maternal smoking during pregnancy and the risk of CBT. The solid line represents point estimates of association between maternal smoking during pregnancy and CBT risk; dashed lines are 95% CIs. Circles are the dose-specific RR estimates. The relative size of each circle is proportional to the inverse variance of the RR.

Using a restricted cubic splines model, we did not find a curvilinear association between parental smoking and the risk of CBT (*P* = 0.619, 0.638, 0.924, and 0.749 for non-linearity, respectively). The summary RRs of CBT for an increase of 10 cigarettes per day were 0.98 (95% CI 0.92-1.04; *P* = 0.506 for linear trend), 1.04 (95% CI 0.98-1.11; *P* = 0.196 for linear trend), 0.95 (95% CI 0.89-1.02; *P* = 0.179 for linear trend), and 1.02 (95% CI 0.96-1.07; *P* = 0.598 for linear trend) for maternal smoking during pregnancy, paternal smoking during pregnancy, maternal smoking before pregnancy, and paternal smoking before pregnancy, respectively (Figures 3 and S2).

### Influence analysis

In the influence analysis, the influence of each study on the pooled RR was examined by repeating the meta-analysis while omitting each study, one at a time. The study-specific RRs ranged from the lowest values of 0.91 (95% CI 0.83-0.99), 1.06 (95% CI 0.94-1.21), 0.91 (95% CI 0.93-0.99), and 1.08 (95% CI 0.99-1.19) to the highest values of 0.99 (95% CI 0.89-1.10), 1.12 (95% CI 0.98-1.27), 0.96 (95% CI 0.85-1.08), and 1.11 (95% CI 1.00-1.24) for maternal smoking during pregnancy, paternal smoking during pregnancy, maternal smoking before pregnancy, and paternal smoking before pregnancy, respectively (Figure S3).

### Evaluation of heterogeneity

For maternal smoking during pregnancy, low to moderate between-study heterogeneity was observed for the pooled RR (I^2^ = 28.2%, 95% CI 1.6%-60.7%) and several subgroup results, including cohort studies (I^2^ = 59.0%), studies conducted in Europe (I^2^ = 65.2%, 95% CI 16.3%-85.5%), Ependymomas (I^2^ = 28.9%, 95% CI 0.0%-73.8%), studies of cases > 300 (I^2^ = 42.2%, 95% CI 0.0%-75.7%), and studies published after 2000 (I^2^ = 50.1%, 95% CI 0.0%-77.7%).

For paternal smoking during pregnancy, there was no obvious heterogeneity between studies, except for Astrocytomas (I^2^ = 52.1%, 95% CI 0.0%-84.2%); for maternal smoking before pregnancy, moderate heterogeneity was observed for studies conducted in Europe (I^2^ = 51.6%); for paternal smoking before pregnancy, no heterogeneity was found in any of the categories.

### Publication bias

**Figure 4:**
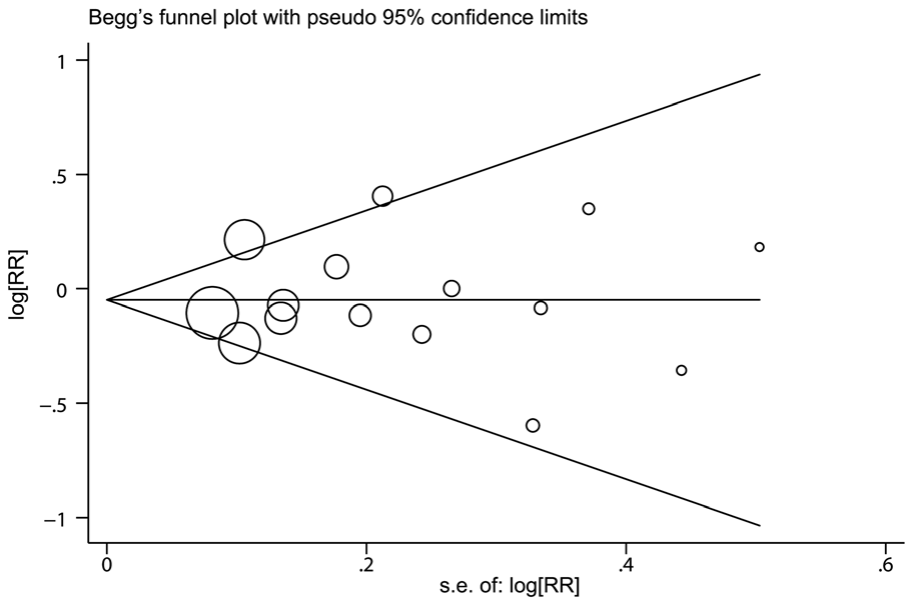
Funnel plot of maternal smoking during pregnancy and the risk of CBT. The area of each square is proportional to the study's weight (inverse of variance).

There was no evidence of significant publication bias according to the Begg and Egger tests (Figure 4, Begg, *P* = 0.528, Egger, *P* = 0.790 for maternal smoking during pregnancy; Begg, *P* = 0.348, Egger, *P* = 0.420 for paternal smoking during pregnancy; Begg, *P* = 0.764, Egger, *P* = 0.610 for maternal smoking before pregnancy; Begg, *P* = 0.368, Egger, *P* = 0.189 for paternal smoking before pregnancy).

## Discussion

In this systematic review of epidemiological studies, no clear relationship was found between parental smoking and the risk of CBT. Similar results were obtained in dose-response analysis and stratified analysis. Although some of the summary RRs (maternal smoking before pregnancy and paternal smoking before pregnancy) were borderline significant, the magnitudes of these associations were quite modest and within the range in which various sources of bias could explain them. Our findings were based on a total of 17 studies (including over 5,000 cancer cases) without obvious heterogeneity and publication bias. However, limited data were available for certain subgroups (e.g., cohort studies, studies conducted in Asia). Therefore, these results should be interpreted with caution.

Low to moderate between-study heterogeneity was observed for several pooled RRs and subgroup results. For example, low between-study heterogeneity was observed (I^2^ = 28.2%, 95% CI 1.6%-60.7%) for maternal smoking during pregnancy, which was not surprising given the differences in study design, characteristics of populations, histological subtypes, and adjustment for confounding factors. Influence analysis suggested that after omitting some specific studies, the pooled RRs of remaining studies became significant, which indicated that some combined RRs of this meta-analysis were not very steady. For example, the omission of the study conducted by Brooks et al [12] led to a significant inverse association between maternal smoking during pregnancy and CBT risk. This may be because Brooks et al's study is a prospective cohort study, which included large samples (1,441,942 Swedish births) and reported a significant positive association between maternal smoking during pregnancy and CBT risk [12].

Currently, a variety of genetic syndromes, including NF1, NF2, TSC1, TSC2, and VHL, have been causally linked to CBT [38]. However, the environmental risk factors of brain tumors have not been fully established. The only recognized factor is exposure to ionizing radiation, which has been widely reported as significantly increasing the risk of CBT [39], [40]. Other environmental factors, such as cured meats, certain viruses (e.g., JC virus, SV40, etc.), parental heat exposure before pregnancy and fertility treatment, have shown inconsistent associations with CBT [40]-[42]. Although the relationship between parental smoking and CBT risk is biologically plausible, the epidemiological data are complex. Meta-analysis is a useful tool for revealing trends that might not be apparent in individual studies. Using this method, our study doesn't support that parental smoking is an environmental risk factor of CBT.

The largest number of available studies on a specific type of parental smoking was for maternal smoking during pregnancy (n = 16). Consistent with a previous meta-analysis published in 2002 [43], our meta-analysis also found no association between maternal smoking during pregnancy and the risk of CBT. Previous studies have reported that maternal smoking during pregnancy is a possible risk factor for stillbirth [44], child overweight [45] and childhood NHL [46] but not for childhood HL [46], leukemia [47] or testicular cancer [48]. Therefore, maternal smoking may have different effects on offspring through multiple mechanisms, specific and non-specific.

Our meta-analysis also explored the relationship between paternal smoking before and during pregnancy and the risk of CBT. A relationship between paternal smoking and CBT risk is biologically plausible. Linschooten et al reported that paternal smoking could affect the chance of heritable mutations in unstable repetitive DNA sequences [49]. The study of Laubenthal et al also supported that cigarette smoke was a human germ cell mutagen [50]. Additionally, paternal smoking may play a role through the mother's passive exposure to secondhand smoke during pregnancy. Previous studies found that certain compounds in environmental tobacco smoke may pass through the placental barrier and interact with fetal DNA, resulting in DNA damage and mutation [51], [52]. However, the epidemiological evidence on this topic is very controversial. Our meta-analysis, including all the published studies, doesn't support a link between paternal smoking before and during pregnancy and the risk of CBT.

Overall, our meta-analysis did not support the relationship between parental smoking and the risk of CBT, regardless of the source of parental exposure. These similar results between maternal and paternal smoking before and during pregnancy were consistent with the findings of Milne et al, Hu et al, and Gold et al [24], [30], [33], who also investigated all four types of parental smoking. Clearly identifying and classifying the source of smoke exposure may help conduct unbiased assessments of parental smoking, which will help strengthen the conclusion and provide a comprehensive evaluation.

Our study has several strengths. Our meta-analysis of 17 studies involving a large number of cases and participants enhanced the statistical power to detect potential associations and provided more reliable estimates. A dose-response relationship between parental smoking and the risk of CBT was investigated, which further strengthened the conclusion. Half of the included studies considered several subtypes of CBT, allowing us to conduct separate analyses for these cancer subtypes. The absence of important heterogeneity and publication bias supported the robustness of the study findings.

However, several limitations of our meta-analysis should also be acknowledged. First, in this meta-analysis, the vast majority of the included studies were case-control studies. As mentioned previously, recall bias and selection bias might cause a decrease in quality of smoking exposure data. Mothers of children with CBT may be more reluctant to report harmful events during pregnancy than mothers of healthy children [53]. Therefore, this misclassification may lead to biased or spurious results. In recent years, several studies reported that cotinine measured in the dried blood spots was a reliable and accurate marker of maternal smoking close to the time of delivery [54]–[56]. Therefore, this low-cost and objective method could be adapted in future relevant etiologic studies to overcome a moderate amount of exposure measurement error. Second, a meta-analysis is unable to solve problems with confounding factors that could be inherent in the included studies. Inadequate control of all known confounders can produce bias in either direction, toward exaggeration or underestimation of risk estimates [57]. Although we included the data from the most fully adjusted models, residual confounding cannot be completely excluded as a potential interpretation of the observed findings. Third, the results of this study were mainly based on information from western populations, while only two studies [30], [31] from other populations. Different races may have different genetic backgrounds that may affect CBT risk. Thus to generalize the findings, further study in other populations is warranted.

In conclusion, the results from this meta-analysis suggest that, based on available information, parental smoking is not associated with the risk of CBT. Because our meta-analysis has several limitations and the influence analysis suggests that some of the combined results are not very steady, future large well-designed prospective cohort studies with better exposure assessment are warranted to confirm the findings from our study and provide a higher level of evidence.

## Supporting Information

Checklist S1 **PRISMA checklist.**
(DOC)

Figure S1 **Forest plot of paternal smoking during pregnancy (A), maternal smoking before pregnancy (B), paternal smoking before pregnancy (C), and the risk of CBT.**
(TIF)

Figure S2 **Dose-response analysis of paternal smoking during pregnancy (A), maternal smoking before pregnancy (B), paternal smoking before pregnancy (C), and the risk of CBT.** The solid line represents point estimates of association between parental smoking and CBT risk; dashed lines are 95% CIs. Circles are the dose-specific RR estimates. The relative size of each circle is proportional to the inverse variance of the RR.
(TIF)

Figure S3 **Influence analysis of maternal smoking during pregnancy (A), paternal smoking during pregnancy (B), maternal smoking before pregnancy (C), paternal smoking before pregnancy (D), and the risk of CBT.**
(TIF)
