# Supplementary figures and images for: A Meta-Analysis of Parental Smoking and the Risk of Childhood Brain Tumors
Source: PLoS One. 2014 Jul 24;9(7):e102910. doi: 10.1371/journal.pone.0102910 (PMC4109951; doi:10.1371/journal.pone.0102910)

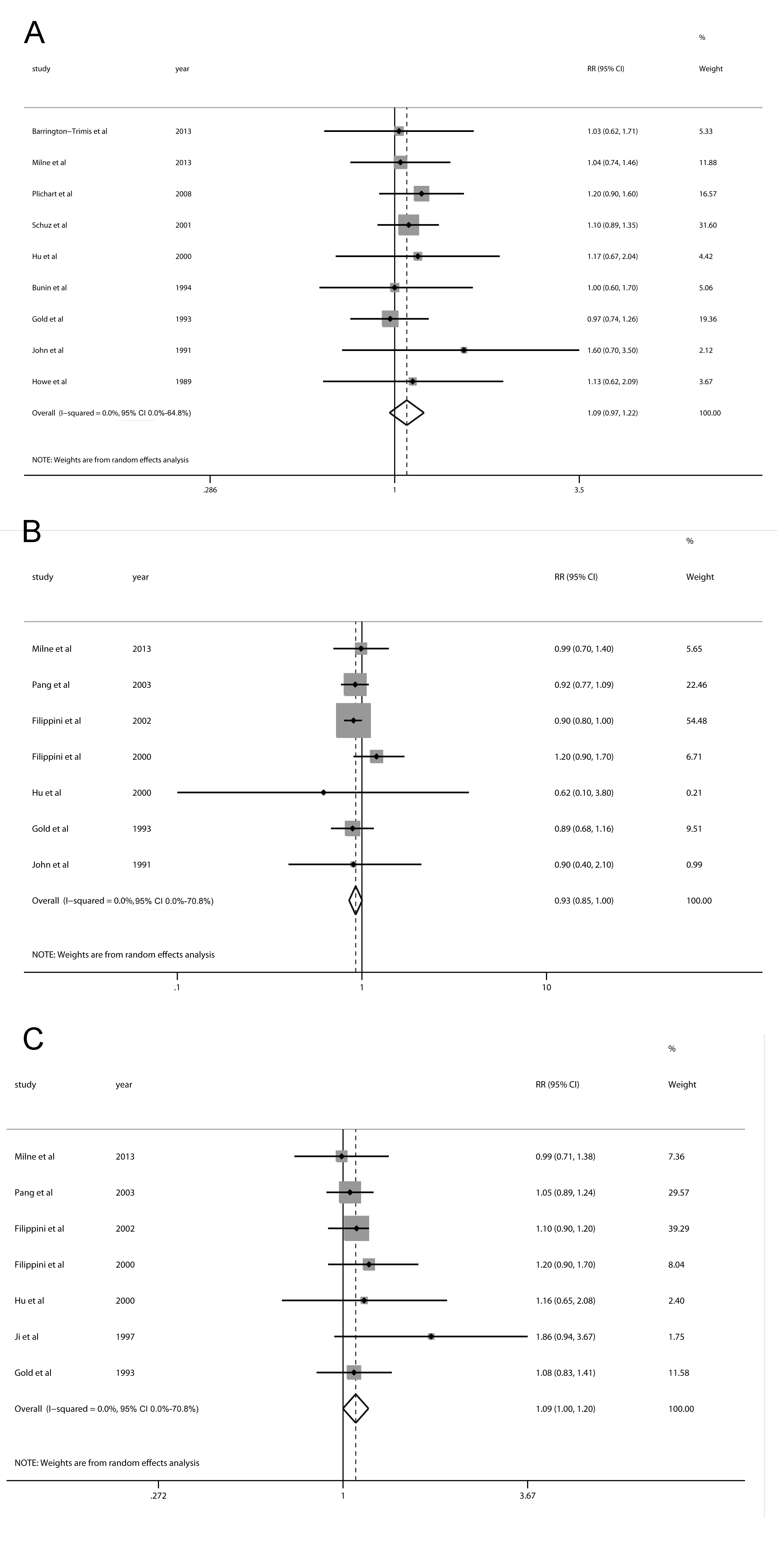

Supplement: Figure S1 — Forest plot of paternal smoking during pregnancy (A), maternal smoking before pregnancy (B), paternal smoking before pregnancy (C), and the risk of CBT. (TIF) [file pone.0102910.s002.tif]

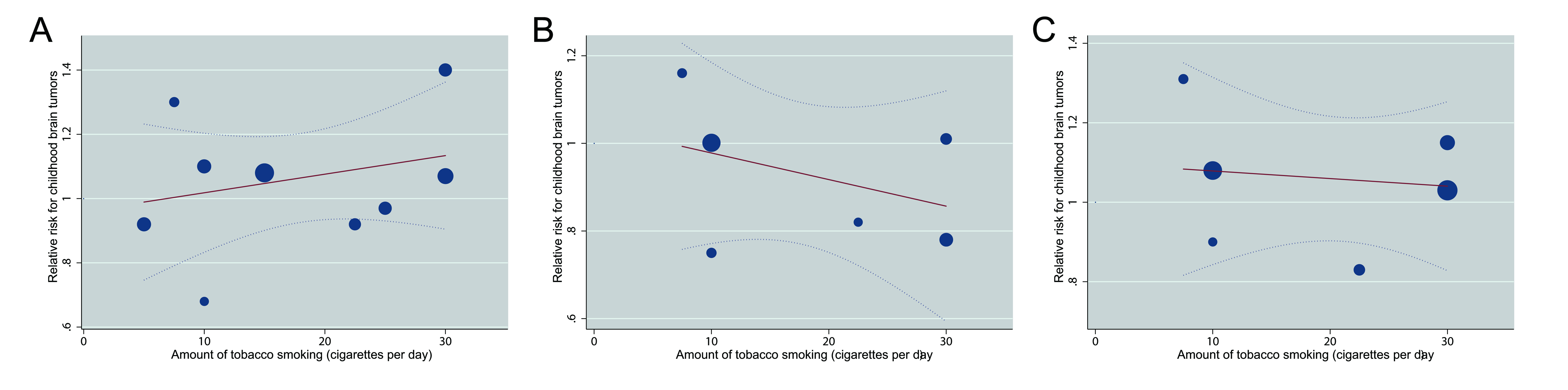

Supplement: Figure S2 — Dose-response analysis of paternal smoking during pregnancy (A), maternal smoking before pregnancy (B), paternal smoking before pregnancy (C), and the risk of CBT. The solid line represents point estimates of association between parental smoking and CBT risk; dashed lines are 95% CIs. Circles are the dose-specific RR estimates. The relative size of each circle is proportional to the inverse variance of the RR. (TIF) [file pone.0102910.s003.tif]

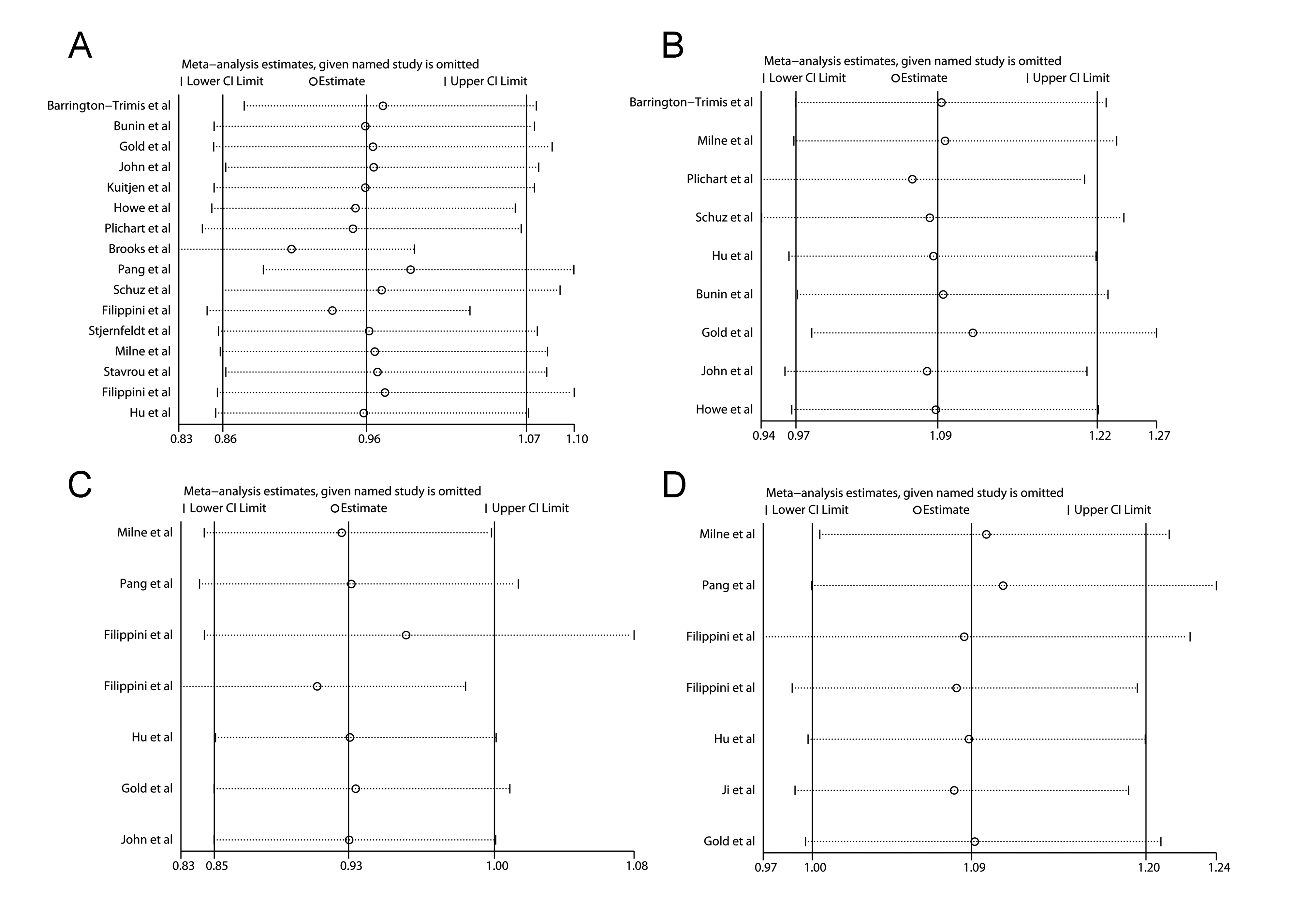

Supplement: Figure S3 — Influence analysis of maternal smoking during pregnancy (A), paternal smoking during pregnancy (B), maternal smoking before pregnancy (C), paternal smoking before pregnancy (D), and the risk of CBT. (TIF) [file pone.0102910.s004.tif]
